# The British Medical Benevolent Fund Guild

**Published:** 1910-06-04

**Authors:** 


### THE BRITISH MEDICAL BENEVOLENT FUND GUILD.

### AN APPEAL TO THE PROFESSION.

A Drawing-room Meeting in support of the funds of the above Guild was held by kind permission of Sir Alfred and Lady Fripp at 19 Portland Place, London, W., on Wednesday, May 25. Mr. Cosmo Bonsor, who occupied the chair, briefly referred to the death of his late Majesty King Edward VII., and said that on such an occasion their first thoughts were with Queen Alexandra and the Royal Family in the great loss we had all sustained. He felt quite confident that all of them, both individually and collectively, had done their best to show, in the words of our King, that they were not standing alone in the great grief that had fallen on the nation. They at first thought of postponing the meeting, but he was quite certain that would have been the very last thing his late Majesty would have wished. He believed that King George V. would be quite as ready as his late Majesty had been to do everything in his power to promote the relief of distress.

The Objects of the Guild.

Mr. Stanley O. Buckmaster, K.C., who moved the main resolution, said: Mr. Chairman and Ladies, if I may use a professional expression, I am here in the position of a *locum tenens*, always a difficult position for a man to occupy, and one which this afternoon I find has added difficulty, since I fear that any shortcomings on my part may hinder the success of the cause my desire to promote which is my only excuse for detaining you here this afternoon. The resolution I have been asked to move is:

> That this meeting pledges itself to do its utmost to support the objects of the British Medical Benevolent Fund Guild.

Those objects are all stated in clear terms in the papers you have before you, but I may just explain those words in a few sentences of my own. There is, as you all know, a fund on foot whose object is the relief of the distress of medical men and their families. The object of this Guild is to support that fund, and to carry its purposes and its objects just a little further. First of all, of course, to obtain the necessary subscriptions for enlarging the purposes and usefulness of the fund itself; and secondly, to do something which it seems to me all may help in doing, even if they are not possessed of the money that enables them to give large subscriptions, that is, to lend the personal assistance of seeing and entering into the wants and troubles of the less fortunate members of this great profession, and seeking to assist them both by personal sympathy and by personal help. Now this Guild is formed for that purpose, and my request to you this afternoon is to support the resolution that I have read, and not only to pledge yourselves to help it, but actually, each one of you, to do all that lies in your power to further such objects, with the purpose of which I am sure you must all be in deep sympathy. Now, after all, it is not a great thing that is asked. It seems to me all I am asking is that you should do something to benefit a class of men who have benefited us.

What the Profession has Done.

The medical profession stands alone and by itself. There is no great trouble, there is no great sorrow of our lives that these men are not called in to witness and to share. It often seems to me that few more pathetic things can be witnessed than the anxiety and suspense with which in the face of some impending catastrophe we listen for their footsteps and hang upon their lips, asking them to do what is often impossible, to set back the hand of the dial, to check the progress of fatal illness, and to arrest the sure and certain foot of Death. And if we make these extravagant demands upon them, I am certain I shall not unsuccessfully appeal to the memory of each one of you here when I say that those demands never fail of meeting with a full response. And if this is true of ourselves, it is true of all the vast area of Society of which, after all, life in the West End only forms a very small, even if it be a glittering, part. In the homes of the very poorest, in the homes of men who are fighting to keep up appearances against a hard struggle in the world, into all places where distress and suffering finds a resting-place, the medical man enters too. And among the best of them—and there are many that reach the best—their foot is never so fleet as when it ascends the staircase of a poor man's home. I always think that you should judge a profession by the best that it produces: firstly, because it shows what the profession is capable of, and secondly, because, after all, the standard of the best men is the standard to which the others try, sometimes imperfectly, but sometimes with success, to reach. Judged by that standard there is no profession in the world comparable to the profession of the practice of medicine. I often think how different it is to the profession to which I have the honour to belong. I have been with great surgeons and great doctors round the wards of hospitals, and I have seen the faces of patients light up as they passed by with the pleasure of their presence and the delight of listening to their voices. There are very few faces light with pleasure at seeing a lawyer if he passes by. It seems to me that it is a great privilege that the medical profession possess to be able, in the pursuit of their profession, to give pleasure and comfort and happiness to the people with whom they are brought into contact. But after all, as you must know well, the struggle is a hard one for many, a hard struggle rendered additionally difficult, and, to my mind, additionally terrifying, by the fact that, however ineffectively it is being waged, appearances have always to be kept up. I can think of nothing more painful than the man who has to keep a brave face and what is called a decent appearance to the world, when he is not quite certain where the money is coming from to pay his butcher's and his baker's bills, whilst at the same time his services are at the call of anyone who likes to come and demand them, even though he often knows that the payment he is to get, even if it is adequate, will be sometimes for a long and indefinite time postponed. There are many instances, as you must know well, where these men render services when there is no hope or prospect of reward.

### The Self-Sacrifice of the Profession.

Think of our great hospitals in London, hospitals which I believe are the finest monuments not only of the philanthropy of our people, but of the public spirit and generosity of the medical men themselves; think of those, and see what time is there ungrudgingly given, and without any hope or recognition of payment, to the very poorest of our people. Think again of an instance which occurs to my mind. Assume a railway accident. The doctor is sent for as a matter of course to come and bind up all the people who are injured; and he comes without a moment's hesitation. He never dreams he is going to get a farthing's payment. They do not send for the lawyers to make their wills, but they send for the doctors to bind up their bodies, and it has never been known that these men have refused to come. The doctor comes perfectly assured that his services will be given without reward, except perhaps in the case where he may have to attend some rich person. There is no doubt, too, that their profession places them day by day and hour by hour in close contact with risks which all of us shun. They possess no special immunity; they are as liable undoubtedly to contract illness and to bring it to their homes as any one of us; yet whatever the risks may be, however deadly, the medical man is there, and incurs the risk much more frequently than we can realise, and it often means the premature loss and the cutting off of the bread-winner of the family. Those are precisely the conditions which this Guild are asked to look into, and those are the cases they are asked to help. I believe I must be speaking to people to whom instances of what I have mentioned are well known. One of the greatest instances of heroism of modern times has always appealed to me, and it is the case of one who with one arm withered to a stump by the use of electrical rays that he was applying for the benefit of his patients, pursued his beneficial work with his remaining arm. In the end that failed too, and the man was thrown out and left without any help whatever except what help could be obtained from the Government and from charitable assistance. That is a shining and notable example; but it is capable of being found in smaller degrees all over the kingdom, and you are asked to use your time, your influence, and your money to help cases such as those. I believe I could not possibly appeal on behalf of any higher cause, and it is that feeling that has led me to urge your acceptance of this resolution in the few sentences with which I have taken up your time.

### How to Help the Guild.

Mrs. Finn, in seconding the resolution, said they all owed a debt of gratitude to the medical profession. Probably there was not one of them who had not at some time or other been glad to incur that debt, and thankful for the help and the comfort given in times of sickness and sorrow. They also knew that a very large portion of that debt could never be repaid, and their only chance of repaying it was to help those who afterwards needed it.

Sir Alfred Fripp also supported the resolution, and said he had not intended to take part in the proceedings, but he had been asked to apologise for the absence of Dr. Russell Wakefield and Dr. Arthur Latham. Dr. Latham, had he been present, would have explained the objects of the Guild, and had intended to point out that it was among the more successful members of the profession that the work must be initiated. In the first instance they had attempted to enlist the sympathies of the senior members of the profession, but now they felt they must also get hold of the junior members. As a result of the invitations sent out for the meeting they had received an offer from one gentleman of an annuity of £20 a year; they had also received a cheque for £10 10s., another for £10, and several cheques for £1 1s. and £2 2s., besides smaller amounts. That was very encouraging, and with the added sinews of war the officials of the Guild would feel themselves justified in making further efforts and expending more money upon the many deserving cases which were brought to their notice.

The resolution was unanimously adopted.

Votes of thanks to Mr. Cosmo Bonsor for presiding, to the various speakers, and to Sir Alfred and Lady Fripp for the use of the room terminated the proceedings.

